# Brief report: bidirectional association of core autism features and cognitive abilities in early childhood

**DOI:** 10.1007/s10803-022-05618-8

**Published:** 2022-08-02

**Authors:** Kelsie McGowan, Daniel Berends, Kristelle Hudry, Giacomo Vivanti, Cheryl Dissanayake, Catherine A. Bent

**Affiliations:** 1 Department of Psychology Counselling and Therapy, School of Psychology and Public Health, La Trobe University, Melbourne, Australia; 2 A.J.Drexel Autism Institute, Drexel University, Philadelphia, USA; 3 Olga Tennison Autism Research Centre, School of Psychology and Public Health, La Trobe University, Melbourne, Australia; 4 Melbourne, Australia

**Keywords:** Core Autism Presentation, Associated Abilities, Developmental Abilities, Cognitive Abilities, Intellectual Disability

## Abstract

We explored associations among the core behavioural features and developmental/cognitive abilities of 155 autistic children, assessed between ages 13–67 months and again around 1-year later to understand predictive directionality. Bidirectional, cross-domain association was apparent, albeit with stronger direction of effect from earlier cognition to later autism features (than vice versa). Exploratory sub-domain analysis showed that early non-verbal developmental/cognitive abilities (only) predicted subsequent *social*- and *restricted/repetitive* autism features, whereas early social features (only) predicted both subsequent *verbal* and *non-verbal* abilities. Although observational study design precludes causal inference, these data support contemporary notions of the developmental interconnectedness of core autism presentation and associated abilities—that behavioural autism features may influence cognitive development, but are likely also *influenced by* an individuals’ cognitive capacity.

Autism Spectrum Disorder (ASD; hereafter, *autism*) reflects a cluster of heterogeneous conditions characterised by social interaction difficulties, alongside restricted/repetitive behaviours (RRBs; American Psychiatric Association [APA], 2013). Current estimates suggest more than one third of people with an autism diagnosis also have an intellectual disability (ID; Centres for Disease Control 2012; Whitehouse et al., 2017) albeit with substantial variability in reported rates (e.g., from 16 to 70%; de Bildt et al., 2005; Fombonne, 2003) potentially attributable to the contemporary recognition of autism as a spectrum-based condition, varying by degree in the expression of behavioural features and associated abilities/difficulties (Constantino & Charman, 2016; Whitehouse et al., 2017). Disentangling the reasons for, and implications of this phenotypic heterogeneity—including that some autistic people present with relatively subtle core behavioural features alongside above-average cognitive abilities, while others present with frank symptoms and ID—is a key concern for contemporary autism research (Georgiades et al., 2013; Kim et al., 2016).

The aetiology underlying co-occurring autism and ID is poorly understood. Some suggest a predictive influence of autism symptoms on intellectual/cognitive development, such that the core features of autism impact a child’s ability and motivation to engage with environmental stimuli, disrupting social learning opportunities important for normative early cognitive development (e.g., Dawson et al., 2004; Su et al., 2021). Empirical support for this proposal includes findings from Vivanti et al.’s (2013) analysis of data from a total sample of 83 young children with autism assessed across one- or two-year follow-up periods. Associations between greater baseline behavioural autism features and subsequently reduced developmental/cognitive gains, suggested that ID might arise from disrupted social learning in the context of an early childhood autism diagnosis.

Another perspective is that the core features of autism likely have some basis in broader cognitive processes (Karmiloff-Smith, 2009; Karmiloff-Smith et al., 2003). For instance, an absence of preferential attention to faces and biological motion (Annaz et al., 2012; Perlman et al., 2011) has been taken as evidence of reduced social motivation, but may reflect more than a simple lack of *interest* in social phenomena—rather, a more fundamental cognitive difficulty that in turn influences a child’s ability to respond to and learn from interaction with others. This suggestion aligns with a ‘compensation framework’; the proposal that the overt presentation of behavioural autism features may reflect, in part, a function of an individual’s ability to *compensate* for their underlying core difficulties using alternative cognitive mechanisms (Karmiloff-Smith, 2009; Livingstone et al., 2018). That is, intellectual/cognitive ability may moderate the apparent extent of autism features, such that individuals with greater cognitive abilities manifest core autism features in less pronounced ways than individuals with cognitive delays or ID. Emerging empirical evidence supporting such an association includes Ben-Itzchak and Zachor’s (2020) recent demonstration that autistic children with greater developmental/cognitive abilities in early childhood presented fewer subsequent autism features in early adolescence. Further, among autistic children grouped as either ‘high compensators’ (with marked Theory of Mind [ToM] impairment despite no overt manifestation of social-affective features of autism) or ‘low compensators’ (with both marked ToM impairment *and* overt social affect features), Livingstone et al. (2018) found strong predictive value for of general intellectual ability, and of specific executive functioning and verbal abilities. Other research also shows reliable predictive value of earlier cognitive abilities for the subsequent development of core autism features, whereby children with better verbal and non-verbal skills have been found to make greater subsequent changes in their behavioural features of autism (e.g., Fein et al., 2013; Gotham et al., 2012).

With both theory and empirical evidence supporting cross-domain influence between core autism features and developmental/cognitive abilities, in both directions, a plausible third suggestion is for a dynamic, *bidirectional* association between characteristics of the autism phenotype. Drawing on data from a large and heterogeneous sample of young children with autism—for whom established, standardised measures were available at two timepoints, on average one year apart—we sought insights into the directionality of association between core autism features and developmental/cognitive abilities by testing *bidirectional* prospective associations. We made the broad prediction that there would be evidence of bidirectional association—with lower baseline autism features predicting greater subsequent developmental/cognitive abilities, and higher baseline developmental/cognitive scores also predicting subsequently reduced scores on a measure of autism behaviours. However, we sought also to extend from past research to test the relative predictive value of each cross-domain association simultaneously, to observe any differences in strength of effect. Further, we also planned a more fine-grained examination of cross-domain associations, separating out key sub-domains for each of autism features (i.e., social affect and restricted/repetitive behaviours), and developmental/cognitive abilities (i.e., non-verbal and verbal abilities). We made no particular predictions here, given previous research on this issue has, to our knowledge, only considered such cross-domain associations unidirectionally, and at the level of higher-order domains.

## Method

### Participants, Procedure and Measures

Data for this study were drawn from a larger evaluation of children with autism enrolled at a community-based early intervention centre between 2010 and 2018, with approval from the La Trobe University Human Research Ethics Committee. In total, 222 children were assessed at service entry (Time 1; 78% male; ages 13–67 months; *M =* 34.63, *SD* = 10.89) and again, on average, one year later (Time 2; interval *M* = 13.50, *SD* = 6.6, range 6.5–34 months). Potentially relevant control factors available within parent-reported demographic/family background information included indicators of child multiplex/simplex status (33/67% respectively), history of developmental regression (65% of cases), and primary caregiver education level as an indicator of family socio-economic circumstance (22% secondary, 42% tertiary, 36% post-graduate).

A measure of core autism features—including presentation across social-affect and restricted/repetitive behaviour domains—was available from the Autism Diagnostic Observation Schedule (ADOS; Lord et al., 2012; with change of edition from ADOS-G to ADOS-2 across the data collection period). Children were administered one of the Toddler Module, or Module One or Two, according to expressive language level and chronological age. ADOS Calibrated Severity Scores (CSS; Gotham et al., 2009) were computed from item-level codes, yielding Total and Social Affect (SA) and Restricted/Repetitive Behaviour (RRB) domain scores, each ranging plausibly from 1 to 10 (with higher scores indicating more behavioural autism features). Measures of developmental/cognitive ability were available from the Mullen Scales of Early Learning (MSEL; Mullen 1995), an assessment suitable for children aged 0–68 months and spanning the four key domains of Visual Reception, Fine Motor, and Receptive and Expressive Language skills. Here, higher raw scores indicate greater developmental/cognitive functioning, and an overall Developmental Quotient (DQ), as well as Non-Verbal and Verbal subdomain DQs were computed as a proxy for IQ (i.e., relevant domain age-equivalence scores expressed as function of child age at assessment * 100; approximating IQ population *M* = 100 and with lower DQ scores suggesting impaired abilities relative to that expected for chronological age).

### Statistical Analysis

A systematic change in service assessment protocol meant Time 2 ADOS data were missing for 65 children (who did not differ on key characteristics from the larger group with complete data available at both timepoints). Only a small amount of missing data (< 5%) was otherwise evident and imputed using the SPSS Expectation Maximation procedure. Two multivariate outlier cases were removed prior to further analysis. We report descriptive statistics for the whole cohort (up to *n* = 222 children) but main analyses for the subsample with ADOS scores available at both timepoints (up to *n* = 155). We examined bidirectional prospective associations between core autism features and developmental/cognitive abilities through path analyses (using AMOS 27.0), using Maximum Likelihood estimation with bootstrapped confidence intervals (2000 samples). Child sex, age, year of service enrolment, and parent-reported history of developmental regression and multiplex/simplex status were considered as potential covariates (though none was significant/retained in the final models).

## Results

Table 1 presents sample descriptive statistics, summarising higher-order and sub-domain ADOS and MSEL scores at each timepoint, and group mean change over time. Average Time 1 ADOS CSS were moderately high but spanned the full possible range of scores. At Time 2, these were similarly varied but with slightly lower mean level. Substantial range was also apparent in individual children’s change scores over time, indicating some had clearly *increased* and others clearly *decreased* their presentation of autism features on this assessment. MSEL DQ scores were similarly varied, with group mean-level scores substantially below 100 at both timepoints, but again spanning very low to well above-average abilities (relative to age). MSEL DQs were slightly higher at Time 2 than at Time 1, but some children again showed very clear developmental progress and others only slower progress relative to chronological age, across the follow-up period.

**Table 1:** Summary descriptive data for behavioural autism features and developmental/cognitive ability scores

	Time 1			Time 2			Difference		
	*n*	*M* (*SD*)	Range	*n*	*M* (*SD*)	Range	*n*	*M* (*SD*)	Range
**Behavioural Autism Features**									
ADOS CSS	203	7.59 (2.02)	1–10	157	6.87 (2.25)	1–10	151	-0.78 (2.12)	-7.0-7.0
SA CSS	201	7.03 (2.04)	1–10	156	6.58 (2.08)	1–10	151	-0.48 (2.34)	-6.0-6.0
RRB CSS	201	8.18 (1.67)	1–10	156	7.82 (1.72)	1–10	150	-0.35 (2.04)	-5.0-4.0
**Developmental/Cognitive Abilities**									
MSEL DQ	212	62.73 (25.86)	15–160	203	66.51 (28.62)	16–128	197	3.63 (15.50)	-58-41
NVDQ	207	70.09 (23.60)	23–164	203	69.49 (26.14)	18–129	194	-0.61 (16.56)	-53-50
VDQ	212	55.97 (30.48)	5-156	203	63.48 (32.42)	5-132	197	7.57 (18.25)	-63-56

ADOS = Autism Diagnostic Observation Schedule, CSS = Calibrated Severity Score, SA = Social Affect domain, RRB = Restricted/Repetitive Behaviour domain, MSEL DQ = Mullen Scales of Early Learning Developmental Quotient, NVDQ = Non-Verbal Developmental Quotient, VDQ = Verbal-Developmental Quotient

### Model 1. Domain-Level Bidirectional Predictive Associations

**Fig. 1:**
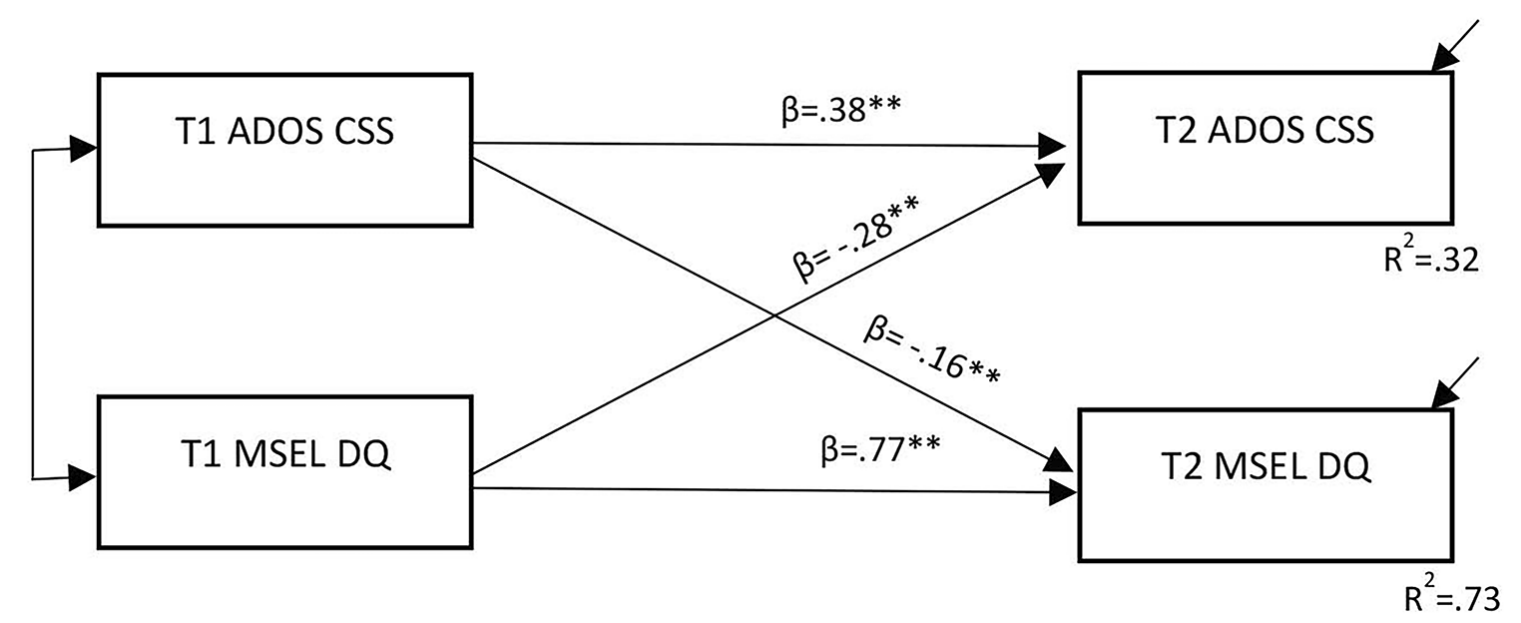
Model of prospective, bidirectional association between developmental/cognitive ability (MSEL DQ) and core autism features (ADOS CSS). *Note*s **p<.001; ADOS CSS = Autism Diagnostic Observation Schedule Calibrated Severity Score; MSEL DQ = Mullen Scales of Early Learning Developmental Quotient

Figure 1 shows the results of multivariate multiple regressions testing the predictive value of Time 1 ADOS Total CSS for Time 2 MSEL Overall DQ, and of Time 1 MSEL DQ for Time 2 ADOS Total CSS. Model fit was deemed to be adequate, with several indices suggesting good fit (CFI = 0.979, SRMR = 0.031, TLI = 0.937), despite other statistically-significant indices—*x*^*2*^(2) = 8.33, *p* = .016 and RMSEA = 0.143 (potentially affected by model complexity; Kenny et al., 2015). All paths were statistically significant, including strong prospective within-domain association for MSEL DQ at Time 1 and Time 2 (*β* = 0.768, 95%*CI* [0.682, 0.834]) and moderate within-domain association for ADOS Total CSS at Time 1 and 2 (*β* = 0.376, 95%*CI* [0.196, 0.537]). Both cross-domain prospective predictive paths were significant—from Time 1 ADOS Total CSS to Time 2 MSEL DQ (*β*=-0.163, 95%*CI* [-0.266, -0.072]) and from Time 1 MSEL DQ to Time 2 ADOS Total CSS (*β*=-0.278, 95%*CI* [-0.431, -0.116]). To determine whether there was a significant difference in the strength of these cross-domain predictive associations, we constrained these paths to be equal, which resulted in a statistically-significant loss of model fit, *x*^*2*^(1) = 11.28, *p* < .001. Hence, the previous ‘best-fit’ model was retained, and explained 73% of the variance in Time 2 MSEL DQ scores from Time 1 ADOS Total CSS (accounting for the continuity of MSEL DQ from Time 1 to Time 2) but only 32% of the variance in Time 2 ADOS Total CSS from Time 1 MSEL DQ scores (accounting for the continuity of ADOS Total CSS from Time 1).

### Model 2. Bidirectional Predictive Associations at Subdomain Level

**Fig. 2:**
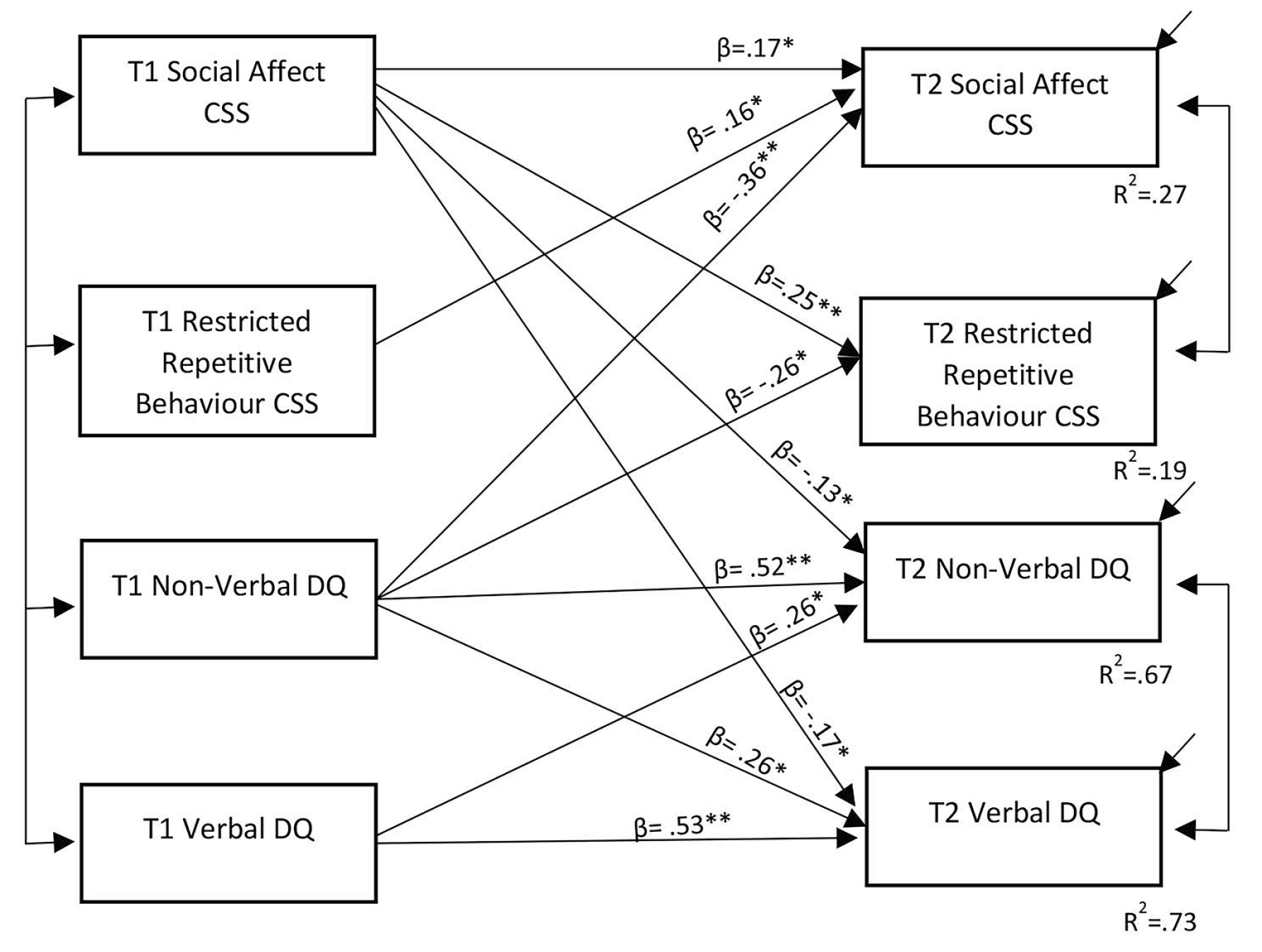
Model of prospective, bidirectional associations between core autism features across Social Affect and Restricted/Repetitive Behaviour subdomains (ADOS CSSs), and developmental/cognitive abilities across Verbal and Non-Verbal domains (MSEL DQs). Notes ^*^p < .05, ^**^p < .001; ADOS CSS = Autism Diagnostic Observation Schedule Calibrated Severity Scores; MSEL DQs = Mullen Scales of Early Learning Developmental Quotients

We also fit a second model to examine bidirectional predictive associations between ADOS CSS and MSEL DQ subdomains (i.e., ADOS SA and RRB CSS; MSEL NVDQ and VDQ scores). For parsimony, non-significant paths were removed from the final model represented in Fig. 2, which showed good fit to the data, *x*^*2*^(9) = 17.236, *p* = .082, with Bollen-Stine bootstrapping (2000 samples) to correct for multivariate non-normality (CFI = 0.991, SRMR = 0.037, TLI = 0.971, RMSEA = 0.077). Small but statistically significant cross-domain associations were evident for higher Time 1 ADOS SA CSS with both lower Time 2 VDQ (*β*= -0.175, 95%*CI* [-0.271, -0.077]) and NVDQ (*β*= -0.133, 95%*CI* [-0.239, -0.020]). No such cross-domain predictive effects were evident, however, for Time 1 ADOS RRB CSS to either MSEL subdomain DQ outcome measure. Moderate, significant cross-domain associations were evident for higher Time 1 MSEL NVDQ with both lower Time 2 ADOS SA CSS (*β*= -0.360, 95%*CI* [-0.500, -0.209]) and RRB CSS (*β*= -0.256, 95%*CI* [-0.413, -0.079]). Again, no such cross-domain predictive effects were evident, however, for Time 1 MSEL VDQ to either ADOS subdomain CSS outcome. The model explained 73% of variance in Time 2 MSEL VDQ and 67% in Time 2 MSEL NVDQ scores from Time 1 ADOS SA CSS (accounting for the continuity of MSEL subdomain DQs from Time 1 to Time 2), and 27% of the variance in Time 2 ADOS SA CSS and 19% in Time 2 ADOS RRB CSS from Time 1 MSEL NVDQ scores (accounting for the continuity of ADOS subdomain CSS from Time 1).

## Discussion

### Evidence for Bidirectional Cross-Domain Associations

Drawing on data from a large, heterogeneous sample of young children with autism for whom scores on established, standardized assessments were available one year apart on average, we sought to clarify the nature of relations between core autism features and broader developmental/cognitive abilities by simultaneously testing bidirectional cross-domain prospective associations. We anticipated that higher developmental/cognitive ability scores would predict subsequently reduced behavioural autism features and vice versa, and sought also to extend from past research to examine the *relative* predictive value of each prospective cross-domain path, as well as to explore effects separately at sub-domain level.

Within our primary model (and our exploratory subdomain model, described below), significant cross-domain prospective association paths were indeed evidenced *bidirectionally*. These data therefore offer support for both accounts that core features of autism may have an influence on downstream developmental/cognitive capacities (e.g., Baghdadli et al., 2012; Vivanti et al., 2013), and equally that broader developmental/cognitive functioning may serve to constrain—or indeed facilitate compensation for—what are considered core features of the behavioural autism phenotype (e.g., Hirosawa et al., 2020). Whereas other related empirical research has tended to appraise one or other direction of influence, we used path analysis to simultaneously appraise bidirectional associations whilst accounting for evident concurrent cross-domain association (of behavioural autism features and developmental/cognitive abilities assessed at the same timepoint) and the consistency/stability of each key domain over time. Preliminary examination of descriptive statistics suggested group mean-level stability, but also evidenced substantial variation in individuals’ change scores on each measure—some children showing substantial increases and others substantial decreases in their scores at test-retest. This variability, along with participant heterogeneity in scores on each assessment at each timepoint—and in terms of other key characteristics (e.g., age and test-retest interval)—give confidence in both the likely representativeness of this cohort from the population of young autistic children, and the fitness-for-purpose of this dataset for the current investigation.

### Exploration of Differences in Relative Strength of Bidirectional Cross-Domain Effects

A key objective of this study was to identify any evidence for stronger effect in one vs. other direction of prospective cross-domain association, informing potential causal developmental pathways underscoring the common co-occurrence of ASD and ID. In our primary model, we identified significantly reduced model fit when we constrained both cross-domain paths to be equal, suggesting better confidence in the original model where the beta value for the path from *earlier* developmental/cognitive ability to *later *behavioural autism features (*β* = 0.28) was larger than that from *earlier* autism features to *later* developmental/cognitive outcomes (*β =* 0.16; a pattern of relative effect strength also seen in our exploratory subdomain analysis, discussed further below).

The overall model presents an apparent contradiction, however; with a greater proportion of the overall variance explained in outcome developmental/cognitive abilities (73%), compared to that for behavioural autism features (32%). This conflicting result–suggesting greater predictive value of earlier developmental/cognitive abilities for outcome behavioural autism features, but greater *overall* variance explained by the model for *outcome* developmental/cognitive abilities–can be reconciled by recognizing the strong within-domain path for early to later developmental/cognitive ability scores, compared to the more modest suggested stability/predictive value in the path for early to later behavioural autism features. We note that both autism behaviours and developmental/cognitive abilities were quantified from standardized measures that are well-established in the field and have been developed to offer fairly stable measurement of latent constructs (i.e., ADOS CSS Lord et al.;^2012^; MSEL DQs norm-referenced and standardized against chronological age, Mullen 1995). Hence, paths suggesting moderate-to-strong within-domain predictive effects, from baseline to follow-up, were unsurprising. Again, however, descriptive data indicated that individual children varied substantially in their extent of change in scores on each measure across the two assessment timepoints. While this dataset was generated in the context of the children’s enrollment within a community-based intervention service (consistently so for all participants), the relatively stronger within-domain path for developmental/cognitive ability scores compared to that for behavioural autism feature scores seems unlikely to be an artefact of the early-intervention experience, as past research evaluating the service-related outcomes have suggested greater benefits for (i.e., more change in) children’s developmental/cognitive abilities than in core features associated with their autism (i.e., pattern of effects opposite to that suggested in the current data; Vivanti et al., 2014).

Notwithstanding considerations around construct measurement and likely generalizability of the current findings given data came from participants with a common intervention service experience, the moderate-to-strong within-domain predictive paths evident in our models were not so strong as to obscure the cross-domain effects of key interest to our investigation. Assuming characteristics of this cohort reflect the heterogeneity of the population of young autistic children, and the available measures are reliable and valid indicators of core autism features and developmental/cognitive ability, our results suggest bidirectional cross-domain influence that favours a stronger directional effect from earlier developmental/cognitive abilities to subsequent core autism features, compared to vice versa.

### Insights from Subdomain Level Analysis

As noted above, results from our exploratory sub-domain level analysis effectively mirrored those from our main domain-level analysis, but suggest some *predictive* specificity. That is, subsequent non-verbal and verbal developmental/cognitive ability indices were predicted specifically by early behavioural autism features in the *social affective* subdomain, and not by those in the restricted/repetitive domain, while outcome social and restricted/repetitive features of autism were specifically predicted by early *non-verbal* developmental/cognitive ability scores, and not by *verbal* ability scores. Again, these findings arose from path analysis in which bidirectional, prospective cross-domain paths were appraised simultaneously, and whilst also accounting for *concurrent* cross-domain associations and prospective within-domain association paths (where, again, paths concerning the within-domain association of earlier and later developmental/cognitive ability scores had larger beta weights than those for within-domain associations for earlier and later behavioral autism features).

Also mirroring the domain-level model, beta weights within the sub-domain level path analysis suggested stronger, prospective predictive effects from earlier non-verbal developmental/cognitive ability scores to later behavioural autism feature scores (social affect *β* = 0.36; restricted/repetitive *β* = 0.26), compared to from earlier social affect features to later developmental/cognitive ability scores (non-verbal *β =* 0.13; verbal *β =* 0.18). However, the model again accounted for a greater proportion of the overall variance in outcome developmental/cognitive abilities (non-verbal 73%; verbal 67%) than for behavioural autism features (social affect 27%; restricted/repetitive 19%), likely due to the higher within-construct consistency of the former.

While a specific pattern suggesting greater predictive value of selected subdomains within the broader categories of developmental/cognitive capacity and behavioral autism features is not unexpected, these findings are not easily reconciled with evidence from past research suggesting, for instance, that verbal abilities are a particularly strong prognostic indicator of later-life outcome in autism (Frazier et al., 2021; Pickles et al., 2014) and that the restricted/repetitive behaviour domain of core autism presentation may facilitate or constrain engagement with environmental learning opportunities (Turner-Brown & Frisch, 2020). Again, to our knowledge the current study is the first to test *bidirectional* cross-domain prospective links between core autism features and developmental/cognitive abilities simultaneously, and our examination at subdomain level was exploratory. Future research should seek to replicate the current findings and to reconcile any robust selective predictive effects with the extant literature.

### Limitations and Future Directions

The current investigation sought to address how core autism features and broader developmental/cognitive abilities influence one another, over developmental time. Observational study design precludes causal inference which would require some experimental manipulation—such as assigning children to receive an intervention designed *either* to modify core features of their autism *or* to enhance their developmental/cognitive abilities, and appraising the relative extent of any downstream cross-domain (and within-domain) benefits. Moreover, our ability to draw strong associative conclusions is also limited by the available sample size, constrained further by the lack of outcome ADOS scores for a sizeable subgroup of the original cohort. We nevertheless note that the cohort represents a reasonably large sample for research of this type, and the heterogeneity in key phenotypic features (as well as age and test-retest interval) characterises the population of interest in ways that are not always true for autism research (see Dykens & Lense 2011) and critical for a strong test of associative hypotheses. We also confirmed that the subgroup with missing outcome ADOS scores was not systematically different from the group retained with complete data, and we evaluated the potential impact of socio-demographic covariates, finding none (and so omitting these from our models for parsimony).

It is somewhat difficult to reconcile the pattern of (a) moderate to strong within-domain effects (i.e., suggesting continuity/stability of developmental/cognitive abilities and core autism behaviours over time), (b) the overall variance explained for each of developmental/cognitive abilities and behavioural autism features as outcome constructs within the models, and (c) the relative strength of effects of cross-domain predictive paths (favouring early developmental/cognitive ability as a stronger predictor of subsequent behavioural autism features than vice versa). Potentially, by the age/developmental stage of children here (i.e., 34 months on average, but varying from 13 months to upward of 5½ years), core autism features and developmental/cognitive functioning are already ‘established’ such that within-domain stability obscures a stronger cross-domain influence that might be evident earlier in life, during key stages of developmental neuroplasticity. In addition to sampling children with autism at younger ages, future research should seek replication across multiple cohorts and could also employ genuinely longitudinal design, examining prospective bidirectional associations between emerging core features of autism and broader developmental/cognitive abilities beyond simple pre-post test design (i.e., across three or more timepoints).
